# Improved Enzyme Replacement Therapy with Cipaglucosidase Alfa/Miglustat in Infantile Pompe Disease

**DOI:** 10.3390/ph16091199

**Published:** 2023-08-23

**Authors:** Lina Fiege, Ibrahim Duran, Thorsten Marquardt

**Affiliations:** 1 Department of General Pediatrics, Metabolic Diseases, University Children’s Hospital Münster, 48149 Münster, Germany; 2 Center of Prevention and Rehabilitation, UniReha, Medical Faculty and University Hospital of Cologne, 50931 Cologne, Germany; ibrahim.duran@unireha-koeln.de

**Keywords:** Pompe disease, glycogen storage disease type II, enzyme replacement therapy, Cipaglucosidase alfa, Myozyme

## Abstract

Pompe disease is a lysosomal storage disorder with impaired glycogen degradation caused by a deficiency of the enzyme acid α-glucosidase (GAA). Children with the severe infantile form do not survive beyond the first year of life without treatment. Since 2006, enzyme replacement therapy (ERT) with Alglucosidase alfa (Myozyme) has been available, which is a recombinant human GAA (rhGAA). Myozyme therapy has prolonged the life span of affected patients, but many patients showed a continuing, albeit slower, disease progression. A new generation of rhGAA, Cipaglucosidase alfa (Amicus) has a higher content of mannose-6-phosphate residues, which are necessary for efficient cellular uptake and lysosomal targeting. Cipaglucosidase alfa is co-administered with an enzyme stabilizer, Miglustat, which also optimizes the pharmacological properties. In mouse models, the superiority of Cipaglucosidase alfa/Miglustat compared to the previous standard therapy could be determined. Here, we report the disease course of a patient with severe infantile M. Pompe, who showed serious progression even with high-dose standard of care ERT. Changing the therapy to Cipaglucosidase alfa/Miglustat improved respiratory failure, cardiomyopathy, and motor functions significantly. The patient could be weaned from respiratory support and oxygen supplementation. Cardiac function was normalized. Most impressively, the patient, who had lost nearly all motor skills, acquired head control, learned to speak, and could move his wheelchair by himself. Overall, the patient’s clinical situation has improved dramatically with the new ERT.

## 1. Introduction

Pompe disease, also known as glycogen storage disease type II, is a rare, progressive, and fatal lysosomal myopathy. 

It has an autosomal recessive inheritance and is caused by mutations in the human GAA (acid alpha glucosidase) gene on chromosome 17q25.3 (Gen Bank accession number NC_000017.11) [1]. More than 200 disease-associated variants of the GAA gene have been described to date [2]. Depending on the genetic defect, there may be complete absence or decreased activity of the acid α-glucosidase (GAA) (Enzyme Commission number EC 3.2.1.20).

GAA is a naturally occurring lysosomal hydrolase that cleaves glucose from lysosomal glycogen. Glycogen is located in the cytoplasm, but molecules past their lifespan will be degraded inside the lysosome. Due to the reduced or absent activity of α-glucosidase, the metabolism of lysosomal glycogen is impaired, resulting in intralysosomal glycogen accumulation. Glycogen accumulations secondarily impair extralysosomal processes such as cell autophagy and various cellular signal transduction pathways [3]. The metabolic disorder particularly affects cardiac, respiratory, and skeletal muscles, such that patients with the severe infantile form clinically show cardiomyopathy, as well as progressive skeletal and respiratory muscle weakness [4].

Age of manifestation and disease severity correlate with the residual α-glucosidase activity. Three courses of Pompe disease are distinguished. In the infantile form, there is either no or only very low α-glucosidase residual activity (less than one percent). The disease manifests itself within a few months after birth and, without therapy, is usually lethal within the first year of life due to respiratory insufficiency caused by diaphragmatic weakness or left ventricular outflow tract obstruction [5,6]. Common symptoms include delayed motor development, muscular hypotonia, poor drinking, and failure to thrive. Clinically, the patients often show macroglossia, hepatomegaly, and hypertrophic cardiomyopathy [7]. Due to the progressive muscle weakness and the pronounced muscle hypotonia, the children do not reach motor milestones at all or only with delay. Moreover, if left untreated, children lose their acquired motor skills again due to disease progression. 

In the juvenile form of the disease, there is up to 10 percent residual GAA activity. These patients only notice the disease at a later stage due to progressive proximal muscle weakness and delayed motor development. 

In the late-onset form, also known as the adult form, the residual activity of α-glucosidase is even higher. In these patients, the first symptoms, such as exercise intolerance and proximal muscle weakness, often do not appear until adulthood [8].

The diagnosis is confirmed by the measurement of reduced enzyme activity in lymphocytes, dried blood spots, muscle tissue, or fibroblasts. Genetic testing of the GAA gene is recommended to identify disease-causing mutations. 

Various biomarkers can be used for diagnosis and therapy monitoring. The disease severity of Pompe disease correlates with the extent of glycogen deposition and with the tissue damage caused by the deposition of glycogen. Accordingly, serum markers that represent the extent of tissue damage can be used for diagnosis. Commonly used biomarkers include creatine kinase (CK), CK-MB, aspartate aminotransferase (AST), alanine aminotransferase (ALT), and lactate dehydrogenase (LDH). However, these markers are all nonspecific and have limited predictive value for disease prognosis [9].

Another biomarker is an oligosaccharide of 4 glucose residues (Glc4), also known as Hex4. This tetrasaccharide is produced during the amylolytic breakdown of glucose. In small amounts, Glc4 is also detected in the urine of healthy individuals. Increased urinary Glc4 excretion has been observed in various pathologies. These pathologies are associated with either increased storage or turnover of glycogen, such as glycogen storage diseases II, III, and IV [9,10]. Unlike the other biomarkers that reflect the extent of tissue damage, Glc4 levels correlate with muscle glycogen storage [11]. In a clinical study of 208 individuals with infantile-onset Pompe disease, Glc4 levels showed a sensitivity of 94% and a specificity of 84% for Pompe disease [9]. The levels of Glc4 in adults are variable. Overall, the results of the study suggest that patients with the infantile course have significantly higher Glc4 excretion than adult patients [9]. Glc4 can be used as a biomarker for diagnosis and therapy monitoring [11,12]. Studies have shown that patients with the best response to therapy had the lowest Glc4 and CK levels respectively throughout the treatment and study periods [11,12].

In infantile patients, about 1/3 of patients show a good clinical response, 1/3 a moderate response, and the remaining 1/3 a poor clinical response to therapy [13]. Consideration has been given to how ERT can be improved for Pompe patients. Especially lysosomal targeting and the alleviation of autophagic pathology were considered to have potential for improvement. With the aim of improving the response of skeletal muscle to ERT, a new generation of ERT was developed.

**Cipaglucosidase alfa**

This new generation of ERT includes chaperone advanced replacement therapy (CHART), in which Amicus Therapeutics combines a higher phosphorylated rhGAA (Cipaglucosidase alfa) with the enzyme stabilizer Miglustat (an imino sugar derivate of glucose). Treatment with Cipaglucosidase alfa/Miglustat was approved by the EMA on 27 March 2023, as a long-term ERT for the treatment of adults with late-onset Pompe disease in the EU [14]. It has been frequently shown in studies that enzyme preparations with a higher mannose-6-phosphate content achieve greater uptake of GAA into cardiac and skeletal muscle than those with no or low levels of M6P [15]. Amicus exploits this effect for the new ERT. Cipaglucosidase alfa is mainly characterized by a higher mannose-6-phosphate content, with mono- and bis-phosphorylated forms. Both forms, but especially the bis-phosphorylated form, show significantly higher affinity to the mannose-6-phosphate receptor than Alglucosidase alfa [16]. Enhanced skeletal muscle targeting results in increased uptake into myoblasts and improved lysosomal transport. Binding of Miglustat to Cipaglucosidase alfa stabilizes the enzyme, preventing denaturation and the associated loss of activity of rhGAA. Studies have shown that with Miglustat, rhGAA exhibited increased activity in the blood and improved uptake into cells (Appendix A—Further information about coadministration with Miglustat) [17]. In a murine model, the new therapy with Cipaglucosidase alfa/Miglustat had significantly better efficacy compared with the previous standard therapy with Myozyme. The new therapy reduced the primary pathology in the form of intralysosomal glycogen deposition more effectively than Alglucosidase alfa. For example, Cipaglucosidase alfa/Miglustat reduced glycogen accumulation in the quadriceps muscle by 73%, whereas Alglucosidase alfa reduced it by only 28% [18]. The response of the diaphragm was also significantly improved, which is a clinically relevant observation, as many patients suffer from respiratory insufficiency due to diaphragmatic weakness [18]. There was an improvement not only in skeletal muscle response but also in cardiac muscle response (Table 1). 

In histology, it is seen that the new therapy caused a reduction of glycogen not only in cardiomyocytes but also in cardiac vascular smooth muscle cells [18]. 

It is hypothesized that the improved efficacy of therapy with Cipaglucosidase alfa/Miglustat is due to the enhanced lysosomal targeting resulting from the mannose-6-phosphate content. A significantly higher proportion of the mature, processed GAA (76 kDA) was detected in the muscle of GAA-KO mice receiving Cipaglucosidase alfa/Miglustat compared to GGA-KO mice with the standard therapy [18].

The affinity of the GAA for glycogen is approximately 7–10-fold higher in the processed, mature 76 kDA variant than in the 110 kDA precursor variant [19]. This results in more effective glycogen clearance in the cells [17].

Autophagy as well as transport and delivery of the enzyme were also positively affected by the new therapy [20]. In a mouse model, administration of Cipaglucosidase alfa/Miglustat was shown to reduce glycogen accumulation more effectively and significantly decrease autophagic accumulation in contrast to the previous standard therapy [3,18]. The impaired autophagy of the cells is poorly affected by the standard therapy, Myozyme. The impaired autophagy of the cells negatively affects the GAA transport and delivery to the lysosomes, which results in reduced effectiveness of Myozyme [21].

Glycogen accumulates in lysosomes in Pompe disease. Immunohistochemical studies of muscle biopsies with anti-lysosome-associated membrane protein 1 (LAMP-1) showed that Cipaglucosidase alfa/Miglustat can reduce LAMP-1 signaling to near normal levels. This reduction can be achieved in type I and type II muscle fibers. In particular, type II muscles such as the quadriceps muscle were reported to be resistant to standard therapy with Myozyme [21]. It is likely that novel therapy with Cipaglucosidase alfa/Miglustat is capable of reducing/reversing lysosomal pathology regardless of muscle fiber type [18].

Another clinical feature of Pompe disease is the muscle atrophy, which is histologically characterized by a smaller muscle fiber size [20]. In the mouse model, it was found that the current standard therapy caused only a small increase in muscle fiber size, whereas the new therapy showed a significant increase in muscle fiber size (Table 1). The increase in muscle fiber size was associated with an increase in muscle strength [11].

In conclusion, the new ERT with Cipaglucosidase alfa/Miglustat was demonstrated in the Pompe mouse model to be superior to the previous standard therapy with Alglucosidase alfa in all studied aspects—intralysosomal glycogen degradation, lysosomal enlargement, autophagy, muscle fiber size, and muscle strength [18].

As part of the approval of Cipaglucosidase alfa/Miglustat, the PROPEL study (NCT03729362) was conducted. A randomized, double-blind, Phase 3 study of participants aged 18 years or older with late-onset Pompe disease, who had either been treated with Myozyme for at least two years or were not receiving enzyme replacement therapy, was carried out. Participants received either Cipaglucosidase alfa (20 mg/kg) plus Miglustat or Myozyme (20 mg/kg) plus Placebo once every two weeks for 52 weeks. The primary endpoint was the change in 6-min walk distance from baseline to week 52. At week 52, the mean change from baseline in 6-min walk distance was 20.8 m in the Cipaglucosidase alfa/Miglustat group versus 7.2 m in the Myozyme plus Placebo group [22] (Table 1). Through this study, it was demonstrated that clinical endpoints can be positively affected by the new ERT.

Here we report what we believe to be the first clinical application of the new enzyme replacement therapy in a pediatric patient with infantile Pompe disease worldwide. In clinical use, Cipaglucosidase alfa/Miglustat in this patient appeared to be clearly superior to the standard ERT.

**Table 1:** Comparison of Myozyme and Cipaglucosidase alfa/Miglustat.

	Myozyme	Cipaglucosidase Alfa/Miglustat
Glykogen reduction (%) *		
- M. quadrizeps	27.5 ± 19.5	72.9 ± 11.5
- Heart	45.2 ± 9.4	84.9 ± 5.7
Muscle fiber size M. quadrizeps (µm) *^1^	32 ± 1.6	37.2 ± 2
Mean change from baseline in 6-min walk distance (m) *^2^	7.2	20.8

* Male GAA-Ko mice received 2 biweekly administrations of 20 mg/kg bw Myozyme or 20 mg/kg bw Cipaglucosidase alfa/Miglustat. Glycogen levels were measured in tissues collected 14 days after the last administration. To calculate percentage reduction, the amount of Glycogen in the WT was subtracted from average glycogen levels in untreated and treated GAA-Ko mice [18]. *^1^ Assessment of muscle fiber size by using minimum Feret’s diameter [18]. *^2^ LOPD received either 20 mg/kg bw Cipaglucosidase alfa/Miglustat or 20 mg/kg bw Myozyme every two weeks for 52 weeks. The difference in 6-min walk distance from baseline and after therapy was measured after 52 weeks [22].

## 2. Results

### 2.1. Case Report: Course of the Disease before Diagnosis of Morbus Pompe 

The index patient was born spontaneously at 38 + 6 weeks of gestation after pregnancy without complications with a birth weight of 3950 g (99. percentile) and a body length of 52 cm (58. percentile). He is the second child of non-consanguineous parents. 

The postpartum course and the neonatal examinations were unremarkable. Early on, the parents noticed that their child’s motor development was slower and the muscle tone was low compared to the healthy older brother and other children of the same age as the patient. For example, the patient could not turn or lift his head in the prone position at the age of five months. The patient developed weakness in drinking due to inadequate sucking, causing stagnant weight gain. Therefore, the parents presented their son to the doctor at the age of five months.

Thereupon, an inpatient admission to the hospital was made due to the motor development delay and dystrophy (age of 6 months: 66 cm body length—3rd percentile, 6.37 kg body weight—11. percentile, BMI 14.6 kg/m^2^—4th percentile). 

**Figure 1:**
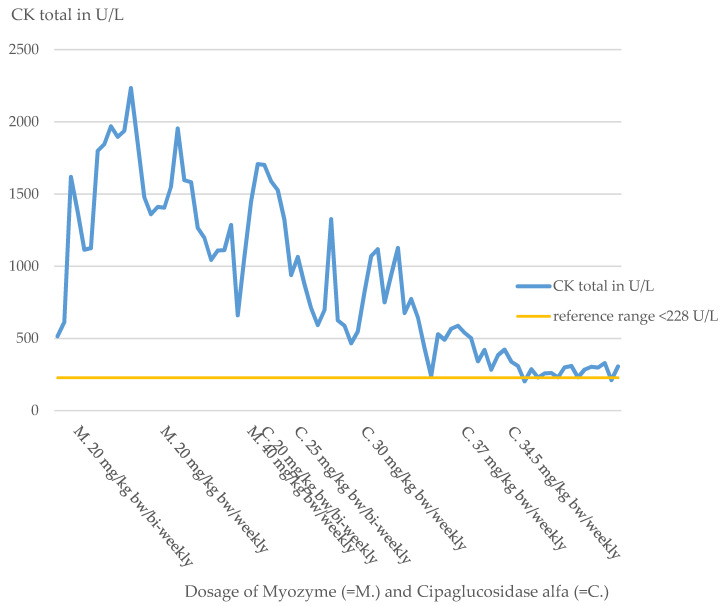
Course of CK-total index patient.

Physical examination revealed muscular hypotonia, decreased reflex status, and pale skin. Laboratory chemistry detected elevations of CK (CK total 515 U/L, reference range less than 228 U/L, Figure 1) and CK-MB levels (CK-MB 41 U/L, reference range less than 24 U/L). ECGs, ophthalmologic examinations, EEGs, and extensive serological tests were performed to complement the diagnosis and were unremarkable. 

Summarizing the results, the diagnosis of an underlying neuromuscular pathology was suspected, and the corresponding genetic diagnostics were initiated. The leading suspected diagnosis at that time was spinal muscular atrophy.

Before the arrival of the genetic findings, cardiomegaly was noticed on a chest X-ray obtained because of suspected pneumonia. This raised the urgent suspicion of Pompe disease. This was later confirmed by metabolic diagnostics and genetic testing. 

#### 2.1.1. Metabolic Diagnostics

Significantly decreased activity of acidic alpha glucosidase was detected in lymphocytes and a dry blood test. In lymphocytes, alpha glucosidase was severely decreased at pH 4 (0.7 nmol/h/mg, reference range > 14 nmol/h/mg). This was confirmed in the dry blood examination. There, the activity of alpha glucosidase was significantly decreased at a pH of 3.8 (0.33 nmol/spot∗21 h, reference range 1.5–10 nmol/spot∗21 h). 

#### 2.1.2. Genetic Analysis

DNA sequencing of the acidic alpha glucosidase gene (Gen Bank accession no. NC_000017.11) revealed two heterozygous mutations. In exon 2, a deletion c.525delT (p. E176Rfs*45), known in the literature to be disease-causing, was found.

This deletion in one base pair (delta T525) on this allele leads to premature termination at nucleotide positions 658–660. This mutation completely prevents the formation of lysosomal alpha-glucosidase [23].

The second mutation, which is also known to cause the disease, is located in exon 18 c.2482-334_2646+39del538. Mutation of the second allele results in an in-frame deletion of exon 18 and adjacent portions of intron 17 and 18. The boundaries of the deletion are marked by an eight nucleotide long tandem repeat (AGGGGCCG) that appears to be critical for the mutation event.

The mutation results in an abnormal 2.3 kb SacI fragment of the human lysosomal α-glucosidase (GAA) gene [24]. The truncated enzyme precursor is produced but degraded prematurely in the endoplasmic reticulum or Golgi complex [25]. Deletion of exon 18 is one of the most common mutations in Pompe disease [23]. 

Genetic testing of the parents confirmed that each parent was heterozygous for one of the diagnosed mutations.

Before starting ERT, it is important to have knowledge about the CRIM (cross reactive immunological material) status. Patients with a CRIM-negative status often show a poor response to therapy, which is particularly attributed to the formation of anti-drug antibodies (ADA). Immunomodulation can be performed to prevent this. Immunomodulation shows the best efficacy when performed before or shortly after initiation of ERT [26]. 

In studies, CRIM status was determined by Western blot analysis on cultured skin fibroblasts. Subsequently, correlation of the determined CRIM status with pathogenic GAA mutations was performed.

One of the most frequently identified mutations with CRIM-negative status in Western blot analysis was the c.525delT mutation (4.8% of CRIM-negative alleles), which is heterozygous in the index patient [27].

The deletion of exon 18 presented on the second allele of the index patient defines a CRIM-negative status for this region since the other allele has a truncation so that the patient is CRIM-negative for exon 18 [27]. Western blot analysis in fibroblast detected GAA bands.

In the past, a poor therapeutic response to Myozyme and early death were observed in another young Pompe patient with a similar genetic constellation (A. Hahn, Giessen, personal communication). This patient also had an allele mutation leading to no endogenous enzyme production and a mutation on the second allele leading to deletion of exon 18 from the GAA gene. The patient’s poor treatment response and death were attributed to antibody formation against ERT (ADA). This case occurred almost 15 years ago, and ADA testing was not available at that time to confirm the suspicion. For the index patient, experienced Pompe clinicians assessed the risk for ADA formation as relevantly high, so that immunomodulation was recommended.

#### 2.1.3. Muscle Biopsy

**Figure 2:**
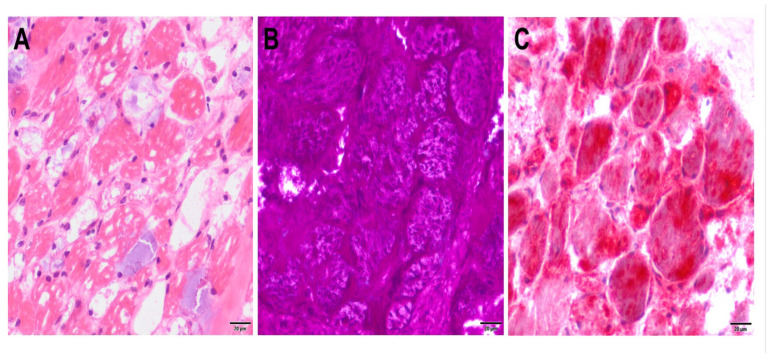
Histopathological and enzyme histochemical features (Scale corresponds to 20 µm—see scale bottom right in the illustrations). On H&E staining, muscle fibers showed a pronounced vacuolar appearance (**A**). There was strong periodic acid–Schiff (PAS) staining, demonstrating large deposits of glycogen in all muscle fibers (**B**) and abundant acid phosphatase activity (**C**).

A muscle biopsy from the pectoralis muscle was taken from the patient at the age of seven months. The HE-section showed highly vacuolated muscle fibers, as well as increased endo- and perimysial connective tissue and a low-grade increase in internally located nuclei. No inflammatory changes were present. PAS staining showed markedly increased glycogen storage (Figure 2). Trichrome staining showed no ragged red fibers or rimmed vacuoles. In the NADH staining, no specific disturbance of the intermyofibrillar oxidative network could be detected. Muscle fibers were difficult to typify using ATPases, but a predominance of type II fibers was observed. The acid phosphatase reaction showed greatly increased lysosomal storage. The morphological and enzyme histochemical findings are typical for Pompe disease.

### 2.2. Case Report: Course of Treatment with Myozyme (Start in the 7th Month of Life–End in the 15th Month of Life)

At the time of diagnosis, cardiac involvement was present in the form of a non-obstructive hypertrophic cardiomyopathy with moderate left ventricular dysfunction (NT-proBNP 14,500–18,000 pg/mL, reference range 12–308 pg/mL; shortening fraction (FS) 21%).

Immunomodulation was initiated based on previous experience in patients with a similar genotype according to the schema of Banugaria et al. [27] with immunoglobulins, rituximab, and methotrexate before starting the ERT. Under the standard dose of Myozyme of 20 mg/kg bw/bi-weekly, food intake remained difficult. The patient was fed completely via a percutaneous endoscopic jejunostomy tube. The patient’s motor development stagnated, and the patient lost already-learned motor skills.

**Figure 3:**
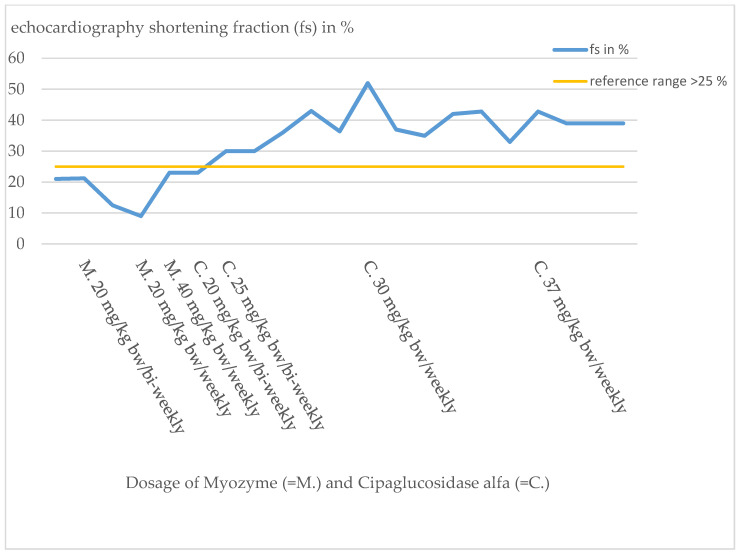
Course of echocardiography shortening fraction (FS) index patient.

Due to the inadequate response to therapy, the dose was doubled just one month after the start of therapy at the age of 7 months in the form of a weekly administration of Myozyme. Under this dose of 20 mg/kg bw/week, the patient showed slight motor improvements, but the cardiological situation deteriorated progressively. Clinically, there were increased signs of heart failure, such as sweating and a reluctance to drink. This impression was also confirmed by echocardiography (Figure 3). The initially obstructive cardiomyopathy progressed to a dilated cardiomyopathy, and a QTc prolongation was noted.

Therefore, just three months after initiation of therapy, the dose was again doubled to 40 mg/kg bw/week, so the patient received a total of four times the standard dose. With the intensification of ERT and cardiac therapy (Table 2), the patient’s condition initially stabilized. Nevertheless, over the following months, the patient’s general condition steadily deteriorated, and the patient decompensated, especially in the respiratory system. 

Due to respiratory insufficiency with tachypnea, increased oxygen demand, and increased secretion because of respiratory muscle failure, non-invasive ventilation became necessary. 

The patient steadily lost motor skills despite dose adjustments of the ERT (Table 3). At the time of contacting Amicus, the patient was 14 months old and had virtually no motor skills left. The patient had no trunk or head control, so the head had to always be supported by an aid or by a person. Movement in the legs was not possible for the patient. The only possibility of extremity movement was minimal movement of the arms while lying down. Movement against gravity was not possible. The patient was also unable to vocalize, so crying was soundless as well. The motor condition also had a drastic effect on the patient’s communication abilities, as the patient was unable to communicate at this time by either sounds or gestures. Facial expressions were also severely limited by the general muscle hypotonia (Supplementary Materials—Video S1). 

**Figure 4:**
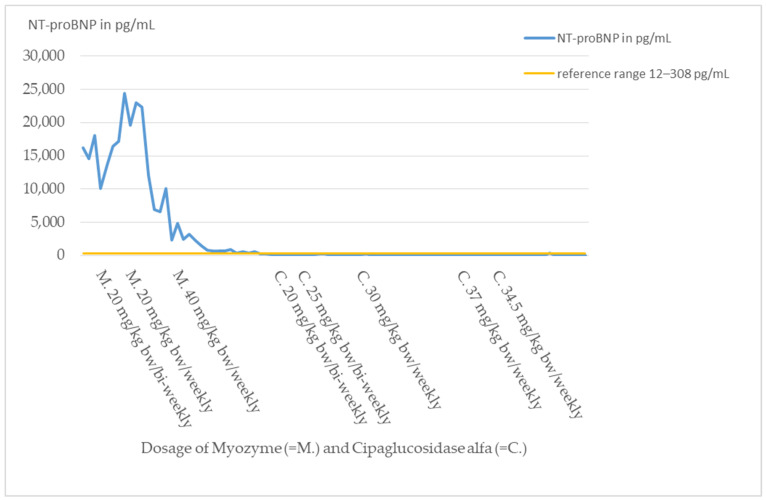
Course of NT-proBNP index patient.

Cardiomyopathy, which normally responds well to ERT with Myozyme, showed a response with decrease in heart failure parameters such as NT-proBNP by laboratory chemistry (Figure 4), but cardiac function measured by shortening fraction (FS) did not show an adequate response (Figure 3). Because of the poor functional response, the patient required increasingly intensive cardiac therapy to avoid cardiac decompensation.

Despite the fourfold dose of ERT and the lack of ADA due to immunomodulation, the patient’s response to therapy was deficient for unexplained reasons. 

At this time, it was assumed that the patient´s clinical course might be fatal. 

### 2.3. Case Report: Course of Treatment with Cipaglucosidase Alfa/Miglustat (Start in the 15th Month of Life–Now)

It was decided to ask Amicus regarding their novel ERT with Cipaglucosidase alfa/Miglustat. At that time, the novel enzyme had been shown to be superior to standard therapy in mouse models of muscle uptake, pharmacokinetics, glycogen degradation, and autophagic accumulations, and human adult studies had been initiated. Amicus agreed to provide the enzyme on a single-patient compassionate use basis. The provision of the drug was made under Amicus’ Expanded Access Program, and parental written informed consent was obtained.

This allowed a switch to the new ERT with Cipaglucosidase alfa and Miglustat after 8 months of insufficient Myozyme therapy response. The patient was 14.5 months old at the time of the therapy switch. The new therapy was initiated with the oral administration of 30 mg of the premedication Miglustat one hour before enzyme administration. Cipaglucosidase alfa was administered at a biweekly interval at a dosage of 20 mg/kg bw.

In the further course of treatment, the dosage of premedication and ERT was repeatedly adjusted and optimized (Table 4).

The parents reported motor progress shortly after the first administration of the new ERT. The patient developed increased facial expressions and muscle strength, which resulted in new motor skills. 

The new ERT significantly improved the cardiac and respiratory situation. The heart failure parameters such as NT-proBNP returned to the normal range (Figure 4). Cardiac function showed a significant improvement (FS 30–43%, Figure 3). With time, the supportive cardiological therapy could be reduced and finally completely discontinued. 

After three months with the new ERT, the patient no longer needed oxygen during the day except in the context of infections. The patient developed a pulmonary infection that required invasive ventilation at times. Because of the need for mechanical ventilation, a tracheostomy was discussed. Ultimately, it was decided that intensification of the ERT would be performed first due to the patient’s good response to therapy so far. Subsequently, 30 mg/kg bw Cipaglucosidase alfa was administered weekly five months after the start of therapy. 

Since the weekly enzyme administration, the doctors and parents have observed constant positive progress in the patient. Only two days after the first weekly administration of Cipaglucosidase alfa, the patient regained his voice.

The respiratory situation improved steadily under the new therapy, so the patient did not need non-invasive ventilation even at night anymore 7 months after the switch of the ERT. After 14 months with the new ERT, the patient no longer required any oxygen at night.

Furthermore, a constantly positive motor development could be observed (Supplementary Materials—Video S2, Appendix B—Figure A1). The patient now showed significantly improved head control and trunk stability. The long-term motor goals include further development of head control and independent turning on the side.

Recently, the patient learned to sit freely cross-legged for a short time. The muscle strength of the patient is now sufficient to move independently in the wheelchair. Communication with the patient has also improved greatly, as he learned to speak more and more words. Due to improved motor skills, the patient can play and enjoy, for example, stacking building blocks or doing puzzles.

To promote motor development further, the index patient participated in the interdisciplinary treatment concept “Auf die Beine” in Cologne. This rehabilitation program is specially designed for children and adolescents with musculoskeletal disorders. In this program, the Gross Motor Function Measure 66 (GMFM 66) is used to quantify the patient’s motor skills. The GMFM 66 is a worldwide-standardized test developed and validated for children with cerebral palsy to measure the development of gross motor function over time [28]. During testing, 66 items are examined, and a score of 0 to 100 points is possible.

**Figure 5:**
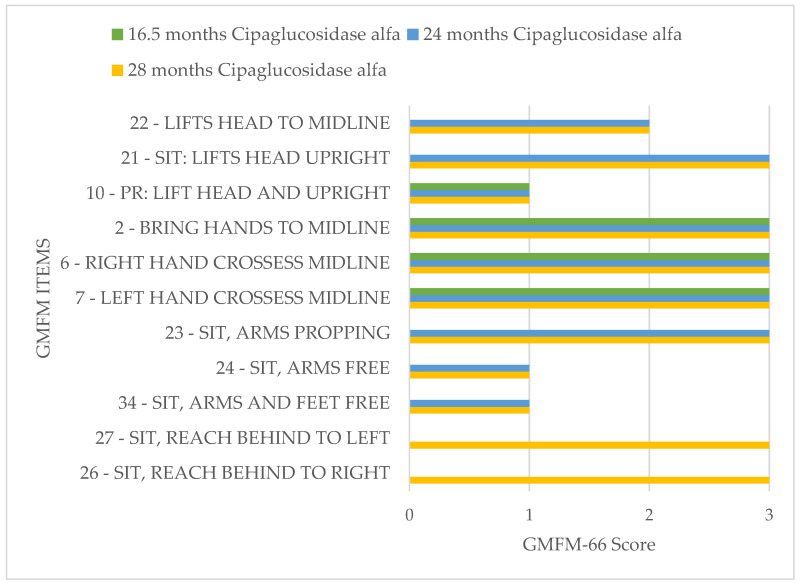
GMFM-66 ITEM Map index patient.

The GMFM 66 has been performed three times on the index patient so far at half-yearly intervals, and he showed a significant improvement in his motor skills over time (Figure 5).

**Figure 6:**
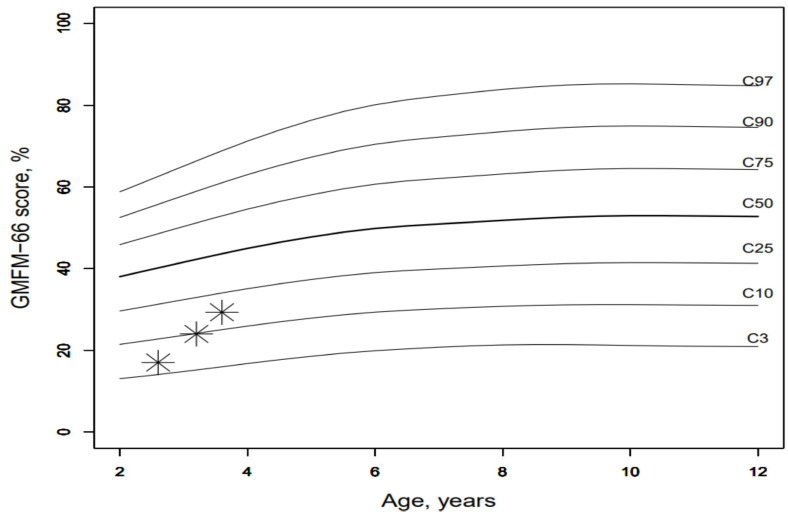
The GMFM-66 trajectory of the case study in relation to GMFM-66 development in children with cerebral palsy.

If we now compare the results of the index patient with those of other children of the same age with cerebral palsy, the index patient shows an unusually good increase in GMFM66 ([29], Figure 6). In the context of dose adjustments, treatment efficacy was higher with high-dose therapy of ERT. With high-dose ERT, faster respiratory and cardiological stabilization, and especially a motor improvement, were noticed. The treatment response of the patient improved at doses above the standard dose evaluated in adults in clinical studies. The patient did not develop any treatment-related side effects, even at higher doses of ERT.

#### Glc4 Analysis

In the index patient, urinary Glc4 has been determined regularly since the switch to Cipaglucosidase alfa in October 2019. At the start of therapy with Cipaglucosidase alfa, the urinary Glc4 value was significantly elevated. The Glc4 levels remained relatively constant under the biweekly administration of a dose of 20 mg/kg bw.

**Figure 7:**
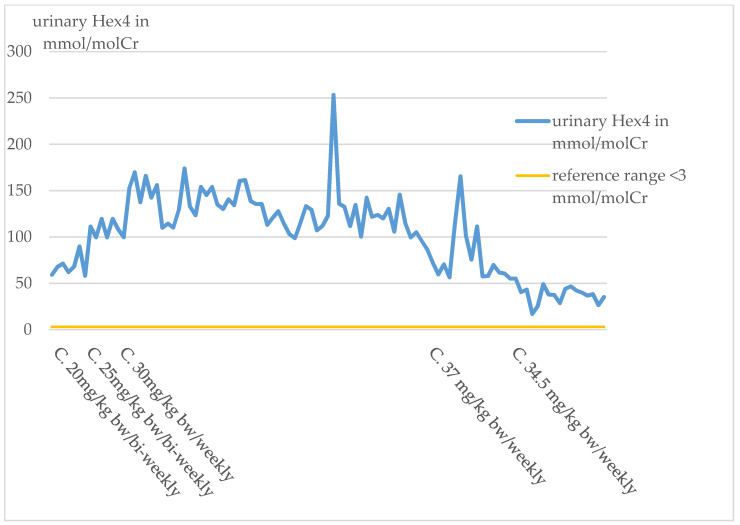
Course of the urinary Hex4 index patient.

An increase in Glc4 levels was observed with the dose increase to 25 mg/kg bw/bi-weekly and also, most notably, with the switch to a weekly administration of 30 mg/kg bw in March 2020. The values stayed at a relatively high level until April 2021. From April 2021, a decrease in the values can be observed (Figure 7).

## 3. Discussion

In this paper, we report a severely affected infantile Pompe disease patient with insufficient response to even high-dose Myozyme therapy, who showed an excellent response to the new ERT with Cipaglucosidase alfa/Miglustat.

The effect of current ERT with Myozyme in Pompe disease is limited in terms of therapeutic response and the impact on the underlying pathology, so only 1/3 of the patients show a good therapeutic response [13]. 

The poor response to therapy is mainly explained by the low mannose-6-phosphate content. The new ERT Cipaglucosidase alfa is characterized by a higher M6P content than Myozyme and, consequently, by improved lysosomal targeting and uptake [15,18].

The patient reported in this paper illustrates that some patients do not respond even to high-dose Myozyme therapy and continue to decline. The patients then deteriorate continuously in motor, respiratory, and cardiac functions until they finally die.

To avert the fatal course of the disease in the case of the index patient due to an inadequate response to therapy, the company Amicus was contacted. Amicus has developed the next generation of ERT for Pompe disease in the form of Cipaglucosidase alfa with the enzyme stabilizer Miglustat. The enzyme stabilizer optimizes the pharmacokinetics of the enzyme. The patient, who was treated on a compassionate-use basis, is now the first pediatric patient to receive the new ERT. 

The patient showed a continuous decrease in his motor skills under the previous therapy with Myozyme (Supplementary Materials—Video S1). Under the new ERT, the patient is learning new motor skills, and his muscle strength is steadily improving (Supplementary Materials—Video S2). The patient required non-invasive ventilation under Myozyme therapy due to respiratory insufficiency caused by respiratory muscle weakness. Meanwhile, the respiratory situation is so stable due to the new ERT that the patient no longer requires any respiratory support or oxygen.

The patient’s cardiomyopathy initially worsened despite Myozyme therapy and additional intensive cardiological therapy. Heart failure parameters, such as NT-proBNP (Figure 4), continued to increase, and cardiac output on echocardiography was destitute, with FS of 10–20% (Figure 3). Intensification of Myozyme therapy at 40 mg/kg bw/weekly improved cardiac performance (FS 23%) and returned heart failure parameters to the normal range.

By switching therapy to Cipaglucosidase alfa, the cardiac situation stabilized and improved significantly. Since the change of therapy, heart failure parameters are permanently in the reference range and cardiac function improved to FS values up to 43% (Figure 3 and Figure 4).

In summary, this patient report demonstrates that the new ERT is superior to the previous therapy not only in the mouse model but also in clinical application. 

It must be clearly stated again that patients with infantile Pompe disease usually die within the first year of life if they receive no treatment. With the currently approved therapy with Myozyme, the patients survive longer, but during disease, many patients become immobile and dependent on the respirator. The current therapy results with Cipaglucosidase alfa raise the hope that the course of the life span can be clearly alleviated by the new ERT and that the patients will have a better quality of life.

Proven biomarkers for therapy monitoring were CK-total as a parameter for the musculature damage, as well as Troponin T and NT-proBNP as heart failure parameters.

In addition, Glc4-levels were regularly assessed in the index patient for therapy monitoring. 

It was striking that the baseline value of Glc4 at the start of therapy with Cipaglucosidase alfa was significantly higher in this patient than in the other study participants with late-onset Pompe [9]. It is assumed that the high initial Hex4-value can be explained by the infantile course and the complex genotype of the patient.

Pompe patients regularly show a decrease in urinary Glc4 levels under both ERT with Myozyme and Cipaglucosidase alfa/Miglustat. While ERT-naive patients usually show an increase in Glc4 levels during the disease [22]. 

The index patient is a special case in this aspect because his values did not decrease under therapy, but increased significantly, especially since the weekly enzyme administration (Figure 7). 

The higher efficacy of the new ERT is considered to be a potential explanation for the rise in urinary Glc4-excretion. The increased efficacy of the new therapy compared with the previous therapy with Myozyme results in increased mobilization of glycogen accumulations in muscle. Accordingly, the increased mobilization of glycogen accumulations also leads to higher Glc4-excretion in the urine. After one year of weekly administration of Cipaglucosidase alfa, the expected decrease in Glc4 can now be observed. It is assumed that this decrease in Glc4-excretion in the urine correlates with a decrease in glycogen accumulation in the muscles.

The optimal individual dose was determined by pharmacokinetic measurements and clinical response. It was much higher than the one used for adult patients.

The fact that the Glc4-values have again increased significantly since March 2020 as a result of the weekly enzyme administration can be interpreted as an approximation of the optimal dosage for glycogen mobilization. This suggests that a higher dosage of the enzyme is associated with better therapeutic success.

After one year of weekly administration of Cipaglucosidase alfa, the expected decrease in Glc4 can now be observed. It is assumed that this decrease in Glc4-excretion in the urine correlates with a decrease in glycogen accumulation in the muscles.

As described in the case report, clinical experience has shown that higher doses of ERT are associated with greater therapeutic success. Studies have also confirmed that a higher dose of ERT, in these studies with Myozyme, has a significantly better effect on the pathological cascade [30]. Therefore, it is reasonable to assume that patients who are currently receiving ERT are often below the optimal dosage. Due to the better clinical outcome of patients under high-dose ERT in studies, experts (European Pompe Consortium) recommend a dosage of 40 mg/kg bw per week of Myozyme for IOPD patients [31]. In the future, it would be desirable if the dose recommendations for ERT were re-evaluated.

Of course, the evidence regarding clinical efficacy of the new ERT by infantile Pompe disease is currently limited, as the patient presented here is the first child with infantile Pompe disease to receive the new therapy. Due to the excellent efficacy of the new therapy in the registration studies, further studies are now being conducted, also in pediatric patients with infantile Pompe disease. The aim is to obtain further study results, and in this way also to substantiate the successes [31] observed to date.

The current dogma regarding enzyme therapy in lysosomal storage diseases is: “what is gone does not come back”.

This dogma was confirmed in the index patient on high-dose standard of care therapy. Despite significant dose increases, the patient’s condition steadily deteriorated.

However, this case report also shows that with the new generation of ERT, this dogma is no longer true. With the novel therapy, the patient has significantly improved cardiologically and respiratorily and regained lost motor skills and even learned new ones.

This case is encouraging for the future and gives hope that the therapy of lysosomal storage diseases will even further improve in the future.

## 4. Materials and Methods

### 4.1. Data Acquisition and Compliance with Ethical Standards

Therapy with Cipaglucosidase alfa/Miglustat was started as part of an individual treatment trial. The collection of data took place in the context of the necessary medical care and because of accompanying knowledge interest. The data collection of the GMFM-66 Score was performed in the context of standard medical care and the data analysis was approved by the responsible ethics committee (Ethics Committee of the University of Cologne (16-269); the register has been registered at http://www.drks.de (accessed on 12 June 2023, DRKS00011331). Written informed consent was obtained before data collection and analysis. 

### 4.2. Enzymatic Diagnostics of Acid Alpha Glucosidase

In the context of metabolic diagnostics, the activity of acid alpha glucosidase was determined in fibroblasts and lymphocytes at pH 4 and 7. Additional indicators were evaluated, such as the activity of GAA with and without inhibitor, as well as the ratio of the activity at pH 3.8 to pH 7.0. 

### 4.3. Genetic Analysis

To confirm the results of the enzymatic diagnostics, a genetic testing of the GAA-gene was performed. DNA was isolated from EDTA blood. The gene encoding acidic alpha-glucosidase is located on chromosome 17q25.2-q25.3 (Gen Bank accession no. NC_000017.11). First, polymerase chain reaction was used to amplify the 19 protein-coding exons including the adjacent intron regions. Subsequently, DNA sequencing and for the detection of deletions and duplications multiplex-ligation-dependent-product-amplification was performed. Because of the negative family history and autosomal recessive transmission of Pompe disease, it was assumed that the mutations by the patient were located on two separate exons. Therefore, genetic testing of the parents was performed. Afterward, the identified mutations were compared with the mutations known to be disease-causing in the Human Gene Mutation Database.

### 4.4. Muscle Biopsy

Within the first year of life, a muscle biopsy from the pectoralis muscle was taken in the context of the implantation of a port catheter. The obtained material was examined by electron microscopy and enzyme histochemistry. For the patient, PAS-, oil red-, trichrome-, NADH-, ATPase-, and acid phosphatase-stains were applied to the sample.

### 4.5. Urinary Glc4 Analysis

The index patient has regular measurement of the urinary Glc4-levels since the change of the ERT to Cipaglucosidase alfa. The Glc4-levels were determined by an established method from Duke University. The analysis of Glc4 is standardized as a butyl-4-aminobenzoate derivative using [13C6] Glc4 as the internal standard (IS). The measurement is performed as ultra-performance liquid chromatography-tandem mass spectrometry (UPLC-MS/MS). The Glc4-values are then normalized to creatinine [32].

## 5. Conclusions

This clinical experience shows that in some cases, if the response to standard therapy with Myozyme is lacking, a switch to the new therapy with Cipaglucosidase alfa/Miglustat should be considered. Furthermore, in addition to drug treatment, interdisciplinary care of patients is important, especially the implementation of adapted but intensive physiotherapy.

## Figures and Tables

**Table 2:** Heart failure therapy plan index patient at 53 weeks of age.

Drug	Dosage	Daily Dosage
Aldactone	25 mg	1-0-0
Captopril	3 mg	1-1-1
Furosemide	3 mg	1-1-1
Hydrochlorothiazide	10 mg	1-0-1
Propranolol	5 mg	1-1-1

**Table 3:** Myozyme therapy plan index patient diagnosis: 01/02/2019—Last Myozyme application: 08/10/2019.

Start of Therapy	Age of the Patient	Dosage	Interval
14/02/2019	29 weeks	20 mg/kg bw	2-weekly
19/03/2019	33 weeks	20 mg/kg bw	weekly
20/05/2019	42 weeks	40 mg/kg bw	weekly

**Table 4:** Cipaglucosidase alfa/Miglustat therapy plan index patient switch of the ERT 15/10/2019—now.

Start of Therapy	Age of the Patient	Dosage Cipaglucosidase Alfa	Dosage Miglustat	Interval
15/10/2019	63 weeks	20 mg/kg bw	30 mg	2-weekly
23/12/2019	73 weeks	25 mg/kg bw	30 mg	2-weekly
04/02/2020	79 weeks	25 mg/kg bw	35 mg	2-weekly
02/03/2020	83 weeks	30 mg/kg bw	40 mg	weekly
18/08/2020	107 weeks	30 mg/kg bw	60 mg	weekly
23/03/2021	138 weeks	30 mg/kg bw	80 mg	weekly
05/04/2021	144 weeks	37 mg/kg bw	80 mg	weekly
26/10/2021	169 weeks	34.5 mg/kg bw	115 mg	weekly

**Figure A1:**
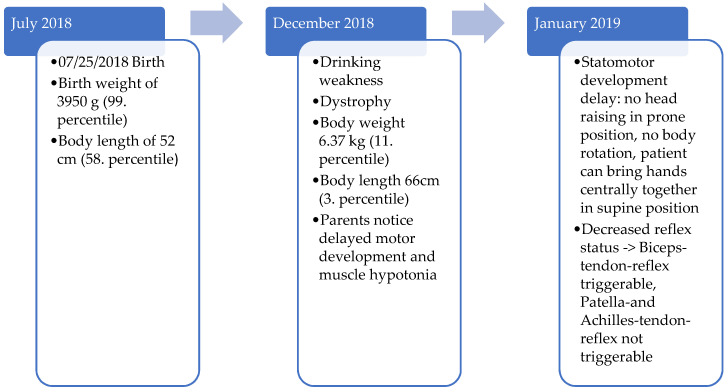

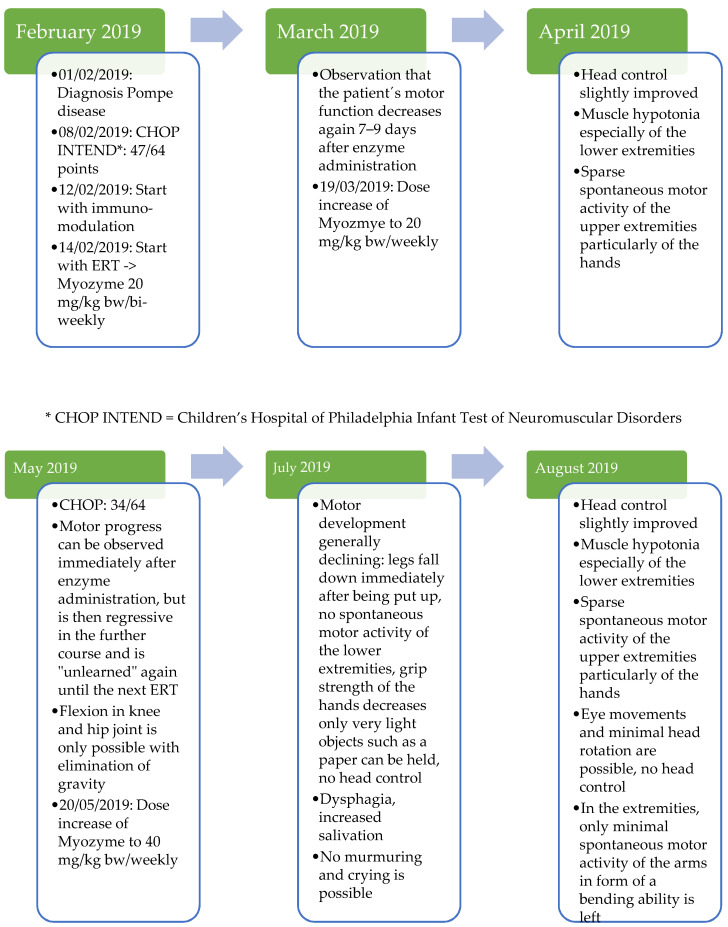

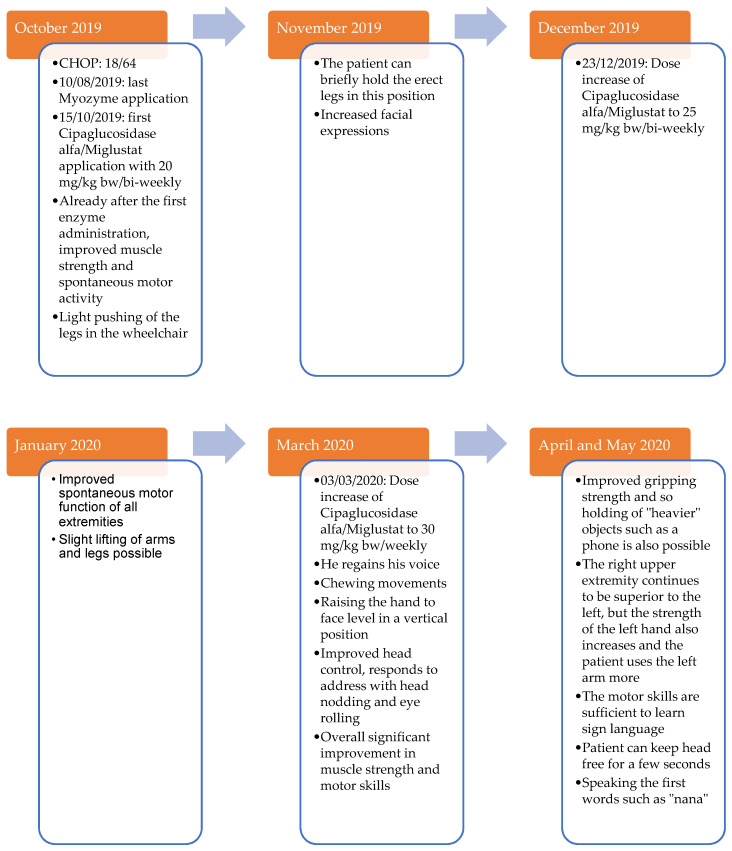

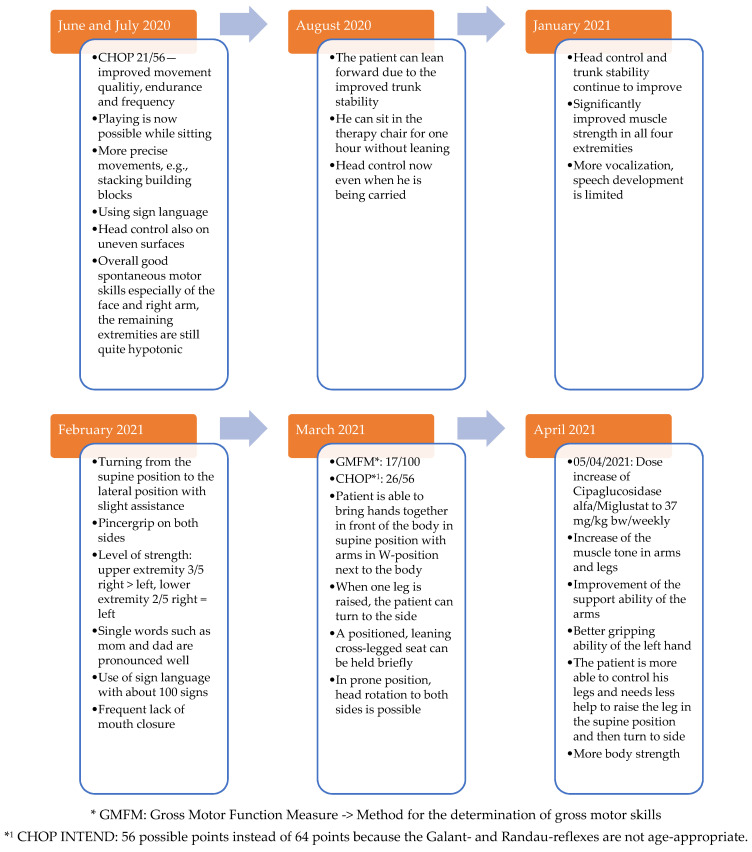

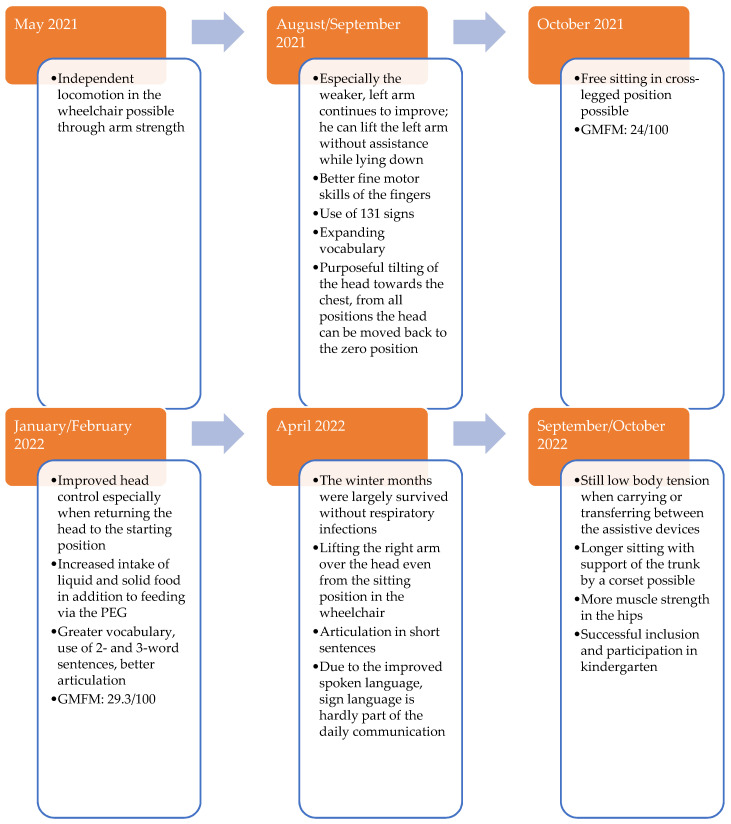

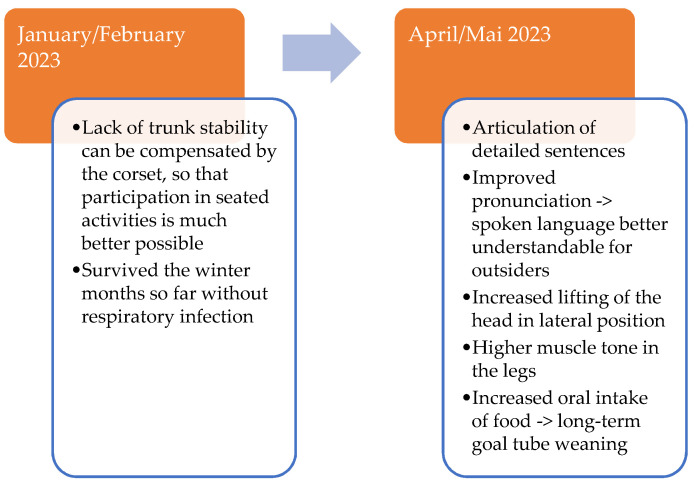
Time course of motor development index patient. Index patient ERT-naïve. Index patient under Myozyme therapy. Index patient under Cipaglucosidase alfa/Miglustat therapy.

## Data Availability

Publicly available datasets of the GMFM-66 Score were analyzed in this study. These data can be found here: [http://www.drks.de accessed on 12 June 2023; DRKS00011331]. The remaining data are contained within the article or supplementary material.

## Supplementary Materials

The following supporting information can be downloaded at: https://www.mdpi.com/article/10.3390/ph16091199/s1, Video S1. Index patient before therapy started with Cipaglucosidase alfa/Miglustat, Video S2. Index patient´s current state under therapy with Cipaglucosidase alfa/Miglustat.

## Appendix A. Further Information about Coadministration with Miglustat

The company Amicus employs the enzyme stabilizer Miglustat in the development of the new ERT to avert premature denaturation and loss of activity of Cipaglucosidase alfa. Miglustat is a small molecule iminosugar administered orally as a premedication prior to the intravenous administration of Cipaglucosidase alfa. The beneficial effect of Miglustat has been substantiated in vivo and in vitro. In a fluorescence-based thermal desaturation assay, Cipaglucosidase alfa was significantly less stable at a pH of 7.4 than at an acidic pH of 5.2. The acidic pH is much closer to lysosomal conditions than the neutral one. When Miglustat was co-administrated at neutral pH, Cipaglucosidase alfa was stabilized, and values were approaching those at acidic pH without Miglustat. Furthermore, in a study conducted on human blood ex vivo at a temperature of 37 degrees, the loss of Cipaglucosidase alfa activity was determined after a duration of 4 h of incubation. Without Miglustat, 63% of the initial activity was lost, whereas only 28% of the activity was lost by coadministration [18].

In clinical practice, this was confirmed. The maximum plasma concentration of Cipaglucosidase alfa (20 mg/kg bw) and Miglustat (260 mg) was determined in patients with LOPD towards the conclusion of the four-hour infusion of ERT. The plasma concentrations decreased biphasically to negligible levels approximately 24 h after the start of the infusion. It was found that Miglustat with Cipaglucosidase alfa increased the area under the concentration-time curve for total GAA protein by about 35% [14].

Miglustat can also achieve a 48% increase in the distribution half-life and a 27% decrease in the plasma clearance of Cipaglucosidase alfa [14].

Excretion of Cipaglucosidase alfa occurs predominantly by proteolytic hydrolysis in the liver. The mean terminal half-life ranged from 1.6 to 2.6 h [33].
